# Intra-operative scleral rupture during 23 gauge pars plana vitrectomy: a case report 

**DOI:** 10.1186/s13256-020-02621-4

**Published:** 2021-01-20

**Authors:** Lalit Agarwal, Nisha Agrawal, Kshitij Aditya

**Affiliations:** 1 Department of Vitreoretina, Biratnagar Eye Hospital, Biratnagar, Nepal; 2 Department of Pediatric Ophthalmology and Strabismus, Taparia Eye Care, Biratnagar, Nepal

**Keywords:** Scleral rupture, Perfluorocarbon liquid, Pars plana vitrectomy, Intra-operative complication, Case report

## Abstract

**Background:**

Use of perfluorocarbon liquid (PFCL) has been increasingly growing as an adjuvant in vitreo-retina surgeries. Some commonly encountered complications with its use include subretinal migration, formation of sticky silicone oil or retained PFCL in vitreous cavity and anterior chamber. Scleral rupture during PFCL injection has a rare occurrence. We report an unexpected event of scleral rupture during PFCL injection and discuss the management challenges faced by the surgeon.

**Case presentation:**

A 66 year indo-aryan male was undergoing pars-plana vitrectomy (PPV) with diagnosis of subtotal rhegmatogenous retinal detachment (RD) with Proliferative Vitreo-retonipathy (PVR)-B. After near total vitrectomy PFCL was being injected and then there was sudden poor visualization of fundus with development of bullous RD and globe hypotony. The surgeon was not able to figure out the cause of hypotony and air was switched on in the infusion cannula. This further complicated the situation resulting in migration of air in the anterior chamber, posterior dislocation of intraocular lens complex, 180° inferior retinal dialysis and ballooning of the conjunctiva which gave a clue of probable scleral rupture. Conjunctival peritomy was performed superiorly and scleral defect was noted. Intraocular tissue incarceration and air leak was visible from the wound. This confirmed scleral rupture during PFCL injection. Repositioning of incarcerated retina was not possible and retinectomy was performed followed by repair of scleral rupture with lots of difficulty in a vitrectomised eye.

**Conclusion:**

PFCL injection, a crucial step of vitreoretina surgery, should be performed slowly with extreme caution maintaining an optimal intraocular pressure to prevent devastating complications like scleral rupture.

## Background

Use of perfluorocarbon liquid (PFCL) has been increasingly growing as an adjuvant in vitreo-retina surgeries. It was first used to reattach retina in eyes with retinal detachment (RD) with giant retinal tear [1]. At present, its use has expanded in complex and recurrent RD, proliferative vitreoretinopathy (PVR) and penetrating ocular trauma. It is also used to stabilize the retina and localize breaks in routine RD, to protect macula and float dislocated lens, to displace subretinal and suprachoroidal hemorrhage.

Scleral rupture during pars-plana vitrectomy (PPV) has a rare occurrence. Some predisposing factors include previously weakened globe due to scleral buckling procedure, preexisting scleral disease, scleral trauma needing repair and high myopia with scleral thinning. Intraoperative globe rupture during PFCL injection step of a planned PPV surgery has rarely been reported. We report this unexpected event during surgery, discuss the diagnostic dilemma and management challenges faced by the surgeon on the operating table.

## Case presentation

A 66 year old indo-aryan male presented with complaint of blurring of vision in right eye (RE) over 7 months. He had undergone cataract surgery 6 years back following which he had good vision in both the eyes. Seven months back he noticed sudden diminution of vision in RE for which he didn’t seek any medical attention and received no treatment. He had no past history of ocular trauma, any other ocular surgery in both eyes or any relevant systemic illness. There was no history of high myopia, diabetes mellitus, hypertension and connective tissue disorder in the patient. He smoked occasionally but didn’t consume alcohol. He was carpenter by occupation. He had two brothers who didn’t suffer from any similar eye problem. At presentation, his vitals were stable. On systemic examination, he had normal vesicular breath sounds bilaterally, heart beat was normal with no added sound, abdomen was soft to palpation and neurological examination was normal. On local examination, his both eyes were pseudophakic, and vision of the RE was hand movement and that of the left eye (LE) was 6/6. His RE had a subtotal rhegmatogenous retinal detachment (RD) with PVR-B (The updated Retina Society Classification) [2] for which vitrectomy was planned. Routine laboratory investigations like CBC, serology, liver and renal functions were performed and were within normal limits.

**Fig. 1:**
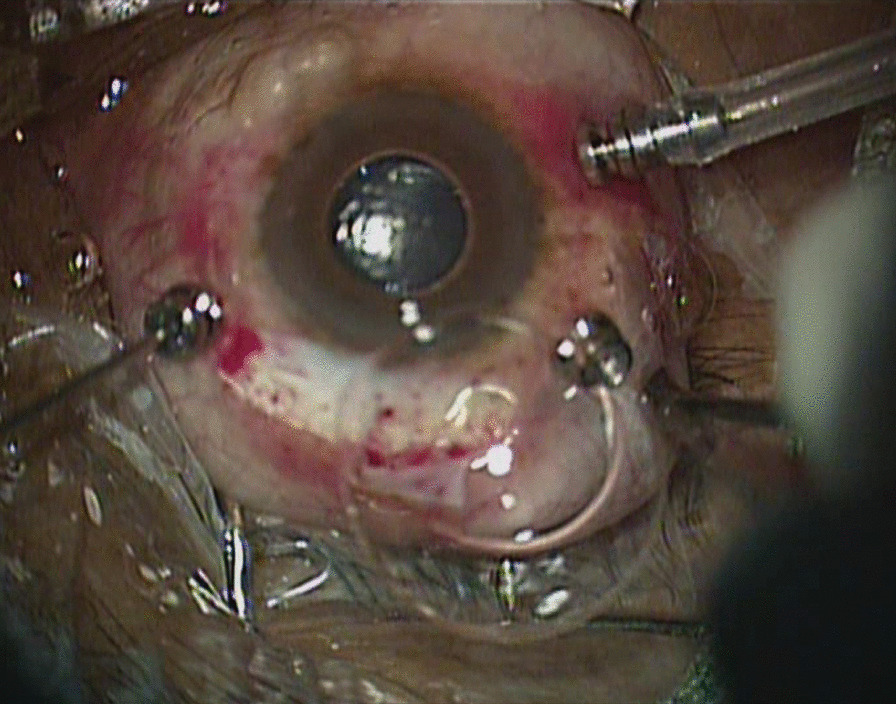
Clinical photo of Right eye of a 66 years old male patient who was undergoing perfluorocarbon liquid injection during pars plana vitrectomy. The photo depicts subconjunctival air and escape of air bubble following conjunctival peritomy.

**Fig. 2:**
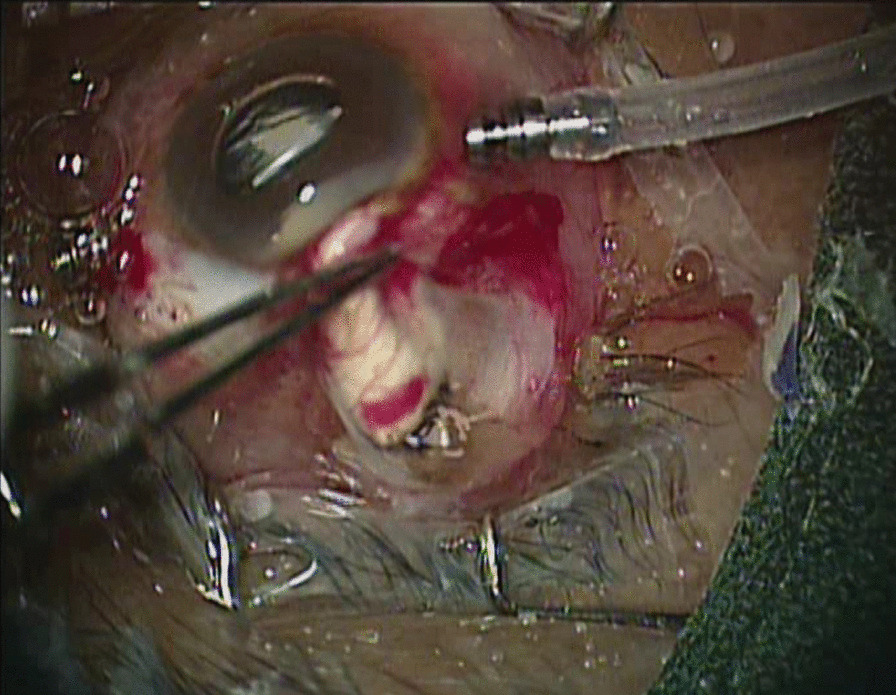
Clinical photo of Right eye of a 66 years old male patient who was undergoing perfluorocarbon liquid injection during pars plana vitrectomy. The photo depicts presence of scleral rupture with intraocular tissue prolapse and air bubble escaping from the defect.

With a diagnosis of subtotal RD, the eye was being operated using 23 gauge vitrectomy systems. After completing near total vitrectomy, perfluorocarbon liquid (PFCL) injection was started using a double bore cannula. During injection of PFCL liquid to flatten the retina, there was sudden change in visibility of the fundus due to increase in haze of the media. There was associated forward protrusion of the eyeball and contact with the binocular indirect ophthalmomicroscope (BIOM) lens. On removing the BIOM, a bullous RD was visible behind the intraocular lens (IOL) and the globe was hypotonus. It was a difficult situation and the surgeon was unable to comprehend what was going on. The cause of hypotony was being explored when the possibility of some mal-functioning of infusion system came into the mind of the surgeon. To overcome the hypotony, air was switched-on in the infusion cannula. This further deteriorated the situation. Air escaped in the anterior chamber, IOL complex dislocated posteriorly, and 180° inferior retinal dialysis was noticed. For some time, the surgeon could not comprehend as to what was going on. Then, ballooning of the conjunctiva was noticed and a provisional diagnosis of scleral rupture was made. Conjunctival peritomy was made superiorly and scleral defect was noticed at 10 o’clock, 10mm behind the limbus extending 9mm in clockwise direction behind the superior rectus muscle. Intraocular tissue incarceration and air leak was visible from the wound (Figures 1 and 2).

Repairing the ruptured globe with retinal tissue incarceration in a vitrectomised eye during vitrectomy was a challenge. If we switched on air in infusion cannula, the turbulence caused at the site of rupture was far more than when balanced salt solution was switched on. Decreasing the infusion pressure would lead to globe collapse. In any situation, repositioning the incarcerated retina was not possible and retinectomy was performed followed by flattening of detached retina with PFCL. This was followed by repair of scleral defect using 9-0 nylon with lots of difficulty. The superior rectus muscle had to be disinserted and reinserted to facilitate scleral repair. Dislocated IOL was removed, vitrectomy was completed and 360° laser retinopexy was performed. RD surgery was then completed and silicone oil was used for tamponade. On first postoperative day visual acuity was hand movement and cornea was edematous. Retina could be poorly visualized and appeared to be attached under oil clinically. He was given topical prednisolone acetate (1%) drop 2 hourly for a week and then tapered gradually over weeks. He was also given topical moxifloxacin (0.5%) eye drop 6 hourly for 4 weeks and topical atropine (1%) eye drop three times a day for 4 weeks. During follow up visits, the retina remained attached under silicone oil and the best corrected visual acuity was 6/36 at last follow-up of 4 months.

## Discussion

We report a case of scleral rupture during PFCL injection while performing pars plana vitrectomy surgery for rhegmatogenous retinal detachment in a 66 year male. This catastrophic complication during PFCL injection has rarely been reported in literature.

PFCLs are serial of fluoro-chemicals which have a characteristic optical clarity, high specific gravity with low surface tension and viscosity [3]. These properties make them ideal for use as an intraoperative tool in vitreoretinal surgery. While injecting PFCL, if one injects it rapidly without submerging the PFCL cannula into the formed bubble, small fish-egg like bubbles may form which can easily migrate subretinally from the retinal breaks. Retained PFCL in subretinal space, vitreous cavity and in anterior chamber are common post-operative complications associated with its use [4]. PFCL in anterior chamber can lead to corneal endothelial damage, cataract formation and glaucoma [4, 5].

20G dual bore cannula was used by Stanley Chang more than 30 years back to inject fluid in the vitreous cavity, simultaneously allowing fluid egress from a vent to maintain IOP. This technique is considered the standard technique of PFCL injection. In the absence of dual bore cannulas, other techniques have been used like using 23-to-25-gauge mismatch or venting via an open pars-plana port or infusion line [6, 7]. If the intraocular fluid fails to exit the eye during PFCL injection, dangerous rise in intraocular pressure (IOP) can occur leading to optic disc hypo-perfusion. It can also lead to anterior ballooning of peripheral retina and formation of iatrogenic tears [7]. However, it has not been reported to cause scleral rupture.

In our patient, the scleral rupture (SR) could have been due to sudden rise in intraocular pressure during PFCL injection, as a result of either rapid injection or due to blocked vent of PFCL cannula. The decision of switching-on of air at the time of hypotony might have further complicated the situation when there was bullous RD. There might have been subretinal migration of infusion cannula, and switching on air in infusion line might have led to inferior retinal dialysis, posterior dislocation of IOL complex, and prolapse of retinal tissue from the scleral defect by the force exerted by air. Ocular hypotony in this situation can also lead to suprachoroidal, subretinal and vitreous hemorrhage.

There are few case reports of scleral rupture that occurred during various steps of intraocular surgery. With respect to cataract surgery, one case of subconjunctival scleral rupture at the time of intraocular lens implantation has been reported [8]. Domínguez Yates *et al*. reported scleral rupture with silicone oil migration into the orbital cavity during silicone oil injection in a case of pathological myopia [9]. Scleral rupture has also been reported during strabismus surgery, scleral buckling surgery, reoperation for failed retinal detachment surgery and during periocular anesthesia [10]. However, we did not come across any report of scleral rupture during PFCL injection in PPV.

The best management plan for such a situation is a matter of debate. If one suspects a misdirected cannula into subretinal or suprachoroidal space, the infusion line can be taken out of lower cannula after plugging the superior sclerotomies. Infusion line can also be fixed to one of the superior cannulas. A light pipe can be inserted into lower (infusion) cannula to confirm cannula's intravitreal position. A very clear message from this case is that air should not be switched on, if one suspects subretinal infusion cannula.

During PFCL injection, apart from submerging the PFCL cannula into the formed bubble and slow injection, one must keep an eye on the optic nerve head to look for signs of raised intraocular pressure. Sudden pulsation of central retinal artery and/or the media turning hazy due to corneal edema should be considered an indication to stop injecting PFCL in the vitreous cavity. Other tips for preventing this kind of complication include checking patency of double bore cannula specially when reusing it.

We also want to emphasize that repairing a scleral perforation prior to vitrectomy is far easier then repairing it in a vitrectomised eye. We could hardly find any guideline/recommendation for repair in such a situation.

## Conclusion

PFCL injection, a crucial step of vitreoretina surgery, should be performed slowly with extreme caution. Maintaining an optimal IOP and observing for intraoperative signs of raised IOP is very important to prevent devastating complications like scleral rupture.

## Data Availability

Not applicable.

## Abbreviations

PFCL: Perfluorocarbon liquid
PPV: Pars plana vitrectomy
RD: Retinal detachment
PVR: Proliferative vitreo-retonipathy
RE: Right eye
LE: Left eye
IOL: Intraocular lens
IOP: Intraocular pressure
SR: Scleral rupture
